# Theranekron Attenuates Inflammation, Oxidative Stress Index, and Apoptosis in Lipopolysaccharide‐Induced Cardiovascular Toxicity: Association With Hypoxia‐Inducible Factor‐1 Alpha‐Related Gene Expression

**DOI:** 10.1002/jbt.71131

**Published:** 2026-10-26

**Authors:** Mehtap Savran, Halil Asci, Orhan Imeci, Furkan Cagri Oguzlar, Ozlem Ozmen

**Affiliations:** ^1^ Department of Pharmacology Faculty of Medicine Suleyman Demirel University Isparta Turkey; ^2^ Department of Emergency Medicine Faculty of Medicine Suleyman Demirel University Isparta Turkey; ^3^ Department of Pathology Faculty of Veterinary Medicine Burdur Mehmet Akif Ersoy University Burdur Turkey

**Keywords:** animal experiment, apoptosis, cardioprotection, endotoxin, inflammation, myocardium

## Abstract

This study aimed to evaluate the possible protective effects of Theranekron, an extract derived from *Tarantula cubensis* venom, on cardiovascular damage induced by lipopolysaccharide (LPS) in an experimental rat model. A total of 32 male Wistar Albino rats were randomly divided into 4 groups: Control, Lipopolysaccharide, Lipopolysaccharide plus Theranekron, and Theranekron alone. Lipopolysaccharide administration caused pronounced histopathological changes in the heart and aorta, including endothelial sloughing, hyperemia, hemorrhage, and infiltration of inflammatory cells. These pathological findings were accompanied by elevated oxidative stress markers, increased tumor necrosis factor‐alpha and inducible nitric oxide synthase immunoreactivity, and increased caspase‐3 immunoreactivity. Additionally, genes associated with the hypoxia‐inducible factor‐1 alpha (HIF‐1α) pathway—such as interleukin 6, signal transducer and activator of transcription 3 (STAT3), and vascular endothelial growth factor—were significantly upregulated in the lipopolysaccharide group. Theranekron pretreatment was associated with partial attenuation of oxidative stress imbalance, of inflammatory and apoptotic immunoreactivity, and of the upregulation of HIF‐1α‐related transcripts. Overall, these findings suggest that Theranekron may offer partial protection against early LPS‐induced cardiovascular injury.

## Graphical abstract

Theranekron alleviates lipopolysaccharide‐induced cardiovascular toxicity by reducing oxidative stress, apoptosis, and inflammation. These protective effects are mediated through the suppression of the HIF‐1α signaling pathway.

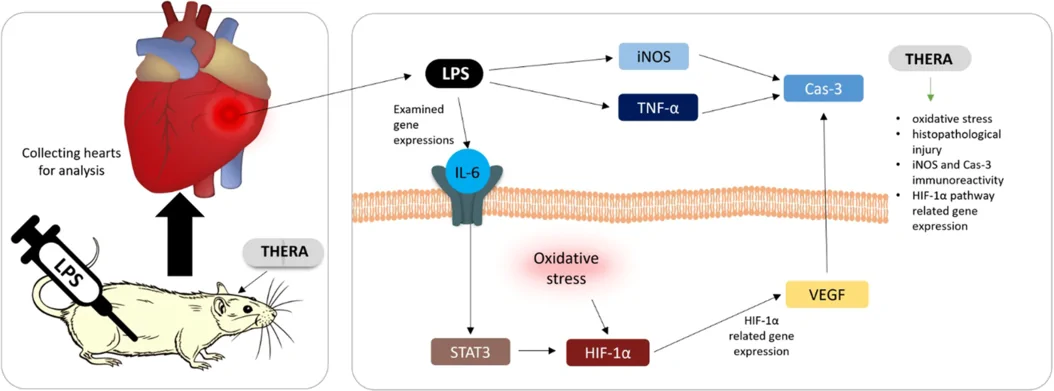

## 1 Introduction

Endotoxemia and the early systemic inflammatory response triggered by lipopolysaccharide can cause considerable cardiac and vascular injury, and current therapeutic approaches remain insufficient to fully prevent this early tissue damage, prompting continued interest in new protective strategies [1, 2].

The heart tissue, responsible for pumping blood throughout the body, along with the vascular structures that facilitate blood delivery to peripheral regions, is particularly susceptible to the detrimental effects of inflammation [3]. At the cellular level, it is known that the receptors located on the affected membrane trigger intracellular mechanisms and cause the damage to expand [4]. These pathways lead to the upregulation of key inflammatory mediators, including tumor necrosis factor‐alpha (TNF‐α), vascular endothelial growth factor (VEGF), and interleukin‐6 (IL‐6). In turn, these cytokines enhance the expression of inducible nitric oxide synthase (iNOS) and caspase‐3 (Cas‐3), which promote apoptotic processes and perpetuate the inflammatory response. This response is further modulated through signaling pathways such as signal transducer and activator of transcription 3 (STAT3) and hypoxia‐inducible factor‐1 alpha (HIF‐1α), both of which are activated by reactive oxygen species and pro‐inflammatory cytokines elevated during oxidative stress, inflammation, and apoptosis [5, 6, 7].

Theranekron, derived from *Tarantula cubensis* (family Theraphosidae), has been reported to have several protective effects in recent publications, but its impact on the cardiovascular system and the aforementioned cellular responses has not been investigated adequately [8, 9, 10, 11]. In this study, we examined whether Theranekron pretreatment is associated with attenuation of cardiac and aortic injury markers and with downregulation of HIF‐1α‐related mRNA expression.

## 2 Materials and Methods

### 2.1 Ethical Approval

The methodology conducted on animals was reviewed and approved by the Experimental Animals Local Ethics Board of Suleyman Demirel University (Approval No: 11.05.2023‐168/05) as it adhered to the ARRIVE 2.0 guidelines.

### 2.2 Animals and Study Design

In the experimental project, 32 male Wistar albino rats (250–350 g) obtained from the Suleyman Demirel University Animal Experimentation Laboratory were used. A 2‐week acclimatization period was implemented prior to the initiation of the experiment. Randomization and blinding methods were employed to minimize bias. Rats were randomly allocated to groups using a random number generator, and outcome assessors were blinded to group assignments. Each group was kept separate, with all rats housed in standard Euro type 4 cages. The animals were maintained under standard temperature and humidity conditions (21°C–22°C and 55% ± 5% humidity) with a 12‐h light and 12‐h dark cycle. Rats were fed ad libitum with standard laboratory chow. The study was conducted with four groups, each consisting of eight animals. All intraperitoneal (i.p.) injections were administered through the right inguinal region.

*Groups:*

1. Control group (*n* = 8): Rats were given 0.2 mL saline subcutaneously (s.c.) and 30 min later 1 mL saline was administered intraperitoneally (i.p.).
2. Lipopolysaccharide (LPS) group (*n* = 8): Rats were given 0.2 mL saline via s.c. route and 5 mg/kg i.p. LPS (L2630‐100 mg, Sigma Aldrich, USA) was applied to induce systemic inflammation 30 min later [12].
3. Lipopolysaccharide + Theranekron group (*n* = 8): Rats were given 0.2 mL Theranekron (1:100 (v/v) dilution in saline, Theranekron, Richter Pharma, Wels, Austria) via s.c. route and 30 min later, 5 mg/kg i.p. LPS was administered to induce systemic inflammation. The dose and administration route were selected based on previous experimental inflammation and LPS‐related injury models in which Theranekron was administered subcutaneously at 0.2 mL of a 1:100 (v/v) dilution and demonstrated anti‐inflammatory effects [13].
4. Theranekron group (*n* = 8): Rats were given 0.2 mL Theranekron via s.c. route and 30 min later 1 mL saline was administered i.p. Theranekron used in this study was the D2 formulation. According to the product composition, 1 mL contains *T. cubensis* alcoholic extract 1:100/D2 1.00 mg and ethanol 96% 402.80 mg.

This design was intended to evaluate the protective effects of Theranekron pretreatment during the early phase of LPS‐induced cardiovascular injury, rather than its therapeutic efficacy after injury onset.

Six hours after the last drug administration, euthanasia was performed under 90 mg/kg ketamine (Ketalar, Pfizer, Türkiye) and 8–10 mg/kg xylazine (XylazinBio, Bioveta, Czech Republic) anesthesia. Following the abdominal incision, blood samples were taken from the inferior vena cava for surgical exsanguination. Half of the heart tissues were placed in tubes and stored at −80°C for biochemical analysis of total oxidant status (TOS), total antioxidant status (TAS), oxidative stress index (OSI), and genetic analysis of IL‐6, STAT3, HIF‐1α, and VEGF expression. Aortic tissues were collected without perfusion immediately after euthanasia to preserve structural integrity for histopathological evaluation. With the other halves of the hearts, they were kept in a 10% buffered formalin solution for histopathologic and immunohistochemical analysis.

### 2.3 Biochemical Analysis

To initiate the experiment, tissues were diluted in phosphate‐buffered saline (10 millimolar Na_3_PO_4_) with a ten‐fold weight/volume ratio, adjusting the pH to 7.4. Subsequently, the tissues underwent homogenization, followed by centrifuging at 10,000 rpm for 10 min at +4°C. The supernatant obtained after centrifugation was utilized to measure the concentrations of tissue TAS and TOS using the colorimetric methods [14]. Results were stated as μmol H_2_O_2_ Equiv/L for TOS, and mmol Trolox Equiv/L for TAS. The OSI was calculated and denoted as TOS/TAS × 100 [15].

### 2.4 Histopathological Method

Following routine tissue processing, fixed aorta and heart samples were embedded in paraffin wax. Sections of 4–6 μm thickness were obtained from the paraffin blocks using a fully automated microtome. After air drying, the sections were stained with hematoxylin–eosin (HE), mounted with coverslips, and examined under a light microscope.

Histological evaluation of myocardial and aortic tissues was performed using a semi‐quantitative ordinal scoring system. In myocardial sections, each lesion parameter‐hyperemia, hemorrhage, inflammatory cell infiltration, and myocardial degeneration/necrosis‐was graded independently on a scale from 0 to 3 (0 = none, 1 = mild, 2 = moderate, 3 = severe) by a pathologist blinded to group allocation as previously described [16] (Table 1). Aortic sections were assessed for endothelial integrity, hyperemia, hemorrhage, and inflammatory cell infiltration using the same semi‐quantitative approach. For each section, multiple representative nonoverlapping microscopic fields were examined and scored.

**Table 1:** Histopathological and immunohistochemical (IHC) scoring criteria for heart tissue.

Score	Description	
	Histopathological findings	Immunohistochemical findings
0	Normal	No staining observed
1	Mild hyperemia, slight disorganization of muscle fibers, presence of a few inflammatory cells and vacuoles	Localized and mild staining
2	Prominent hyperemia, mild hemorrhage, edema, presence of inflammatory cells, and vacuolization of myocytes	Diffuse but mild staining
3	Severe hyperemia, hemorrhage, edema, presence of inflammatory cells, and necrosis of myocytes	Diffuse and intense staining

For immunohistochemical analysis, additional sections were mounted on positively charged slides. Immunohistochemical staining was performed using the streptavidin–biotin method according to the manufacturer's instructions, to assess Cas‐3, iNOS, and TNF‐α expression. Primary antibodies (Abcam Cambridge, UK) were applied at a dilution of 1:100 for Cas‐3 (Anti‐Caspase‐3 antibody [EPR18297] (ab184787)), iNOS (Recombinant Anti‐iNOS antibody [RM1017] (ab283655)) and TNF‐α (Recombinant Anti‐TNF alpha antibody [EPR21753‐109] (ab205587)). After overnight incubation with primary antibodies, sections were incubated with a biotinylated secondary antibody and a streptavidin–enzyme conjugate, followed by visualization with diaminobenzidine (DAB). Negative controls were processed by replacing the primary antibody with antibody diluent. All slides were evaluated by a blinded histopathologist.

### 2.5 Immunohistochemical Evaluation

Immunohistochemical expression was assessed in two complementary ways.

Semi‐quantitative scoring: staining intensity and distribution were evaluated at ×40 magnification using a 0–3 ordinal scale (0 = no staining, 1 = mild focal staining, 2 = moderate diffuse staining, 3 = strong diffuse staining) [16].

Quantitative image analysis: in parallel, positive immunoreactivity was quantified using ImageJ software (NIH, USA) by measuring the percentage area of DAB‐positive staining in standardized fields. Mean values from multiple fields per section were calculated for each animal.

Thus, semi‐quantitative scores were used for ordinal statistical comparison, while ImageJ‐derived measurements were treated as continuous variables. Images were captured using an Olympus CX41 microscope and processed with CellSens Life Science Imaging Software (Olympus Corporation, Tokyo, Japan).

### 2.6 Reverse Transcription‐Polymerase Chain Reaction

Heart tissues were homogenized and RNA was extracted by GeneAll RiboEx RNA Isolation Kit. The quantity and purity of the samples were measured with a UV/Vis spectrophotometer. Complementary DNA (cDNA) was synthesized with an A.B.T. cDNA kit using 1 µg of RNA. Specific primers were found for PCR templates from Primer3 and BLAST (NCBI). Tests were run for potential primer sequences (Table 2). For the quantification of the gene expression levels, A.B.T. SYBR Master Mix with Biorad CFX96 RT‐PCR was utilized and the housekeeping gene was actin B. Then, a reaction mixture was prepared to be 20 µL and put into an RT‐qPCR including thermal cycling setup. Each sample was analyzed in triplicate. The protocol was as follows: an initial denaturation at 95°C for 300 s (1 cycle) and denaturation at 95°C for 15 s, annealing/extension at 55°C for 30 s (40 cycles). The 2^−ΔΔ^ ^Ct^ formula was employed to calculate the relative mRNA levels.

**Table 2:** Primary sequences, product size, and accession numbers of genes.

Genes	Primary sequence	Product size	Accession number
ACTB (housekeeping)	F: CCCGCGAGTACAACCTTCTT	481 bp	NM_031144.3
	R: AACACAGCCTGGATGGCTAC		
IL‐6	F: CACAAGTCCGGAGAGGAGAC	168 bp	NM_012589.2
	R: ACAGTGCATCATCGCTGTTC		
STAT3	F: ATCGCTGAGGTACAATCCCG	412 bp	NM_012747.2
	R: TCTTCGCAGGTTGTGCTGAT		
HIF‐1α	F: GCAACTAGGAACCCGAACCA	251 bp	NM_024359.2
	R: TCGACGTTCGGAACTCATCC		
VEGF	F: TTCGTCCAACTTCTGGGCTC	482 bp	NM_001287111.1
	R: GCTTTCTGCTCCCCTTCTGT		

Abbreviations: ACTB, actin B; F, forward; HIF‐1α, hypoxia‐inducible factor‐1 alpha; IL6, interleukin 6; R, reverse; STAT‐3, signal transducer and activator of transcription 3; VEGF, vascular endothelial growth factor.

### 2.7 Statistical Analysis

Sample size calculation was performed using G*Power 3.1.9.7, based on 4 experimental groups with 8 rats per group (total *n* = 32), assuming *α* = 0.05, power (1 − *β*) = 0.90, and an effect size of 0.75. The primary endpoint was OSI, based on variance estimates from previous studies. Data distribution was assessed using the Shapiro–Wilk test. Statistical analysis was performed according to data type: Ordinal histopathological and semi‐quantitative immunohistochemical scores: analyzed using the Kruskal–Wallis test, followed by Dunn's post hoc test for multiple comparisons. Continuous variables (ImageJ‐based IHC quantification and biochemical parameters including OSI): analyzed using one‐way ANOVA, followed by Tukey's post hoc test for intergroup comparisons. A *p* < 0.05 was considered statistically significant. Analyses were performed using GraphPad Prism 10 (GraphPad Software, USA).

## 3 Results

### 3.1 Biochemical Results

Indicators of oxidative stress, TOS, and OSI values increased statistically significantly in the damage group compared to the control (*p* = 0.002) and Theranekron (*p* < 0.001) groups, while a decrease was observed only in OSI values in the LPS + Theranekron group compared to the LPS group (*p* = 0.036). When the TAS values, which is an indicator of antioxidant activity, were examined, the values in the LPS group were found to be statistically lower than the control (*p* < 0.001) and Theranekron (*p* = 0.015) groups, and in the LPS + Theranekron (*p* = 0.008) group compared to the control group (Figure 1).

**Figure 1:**
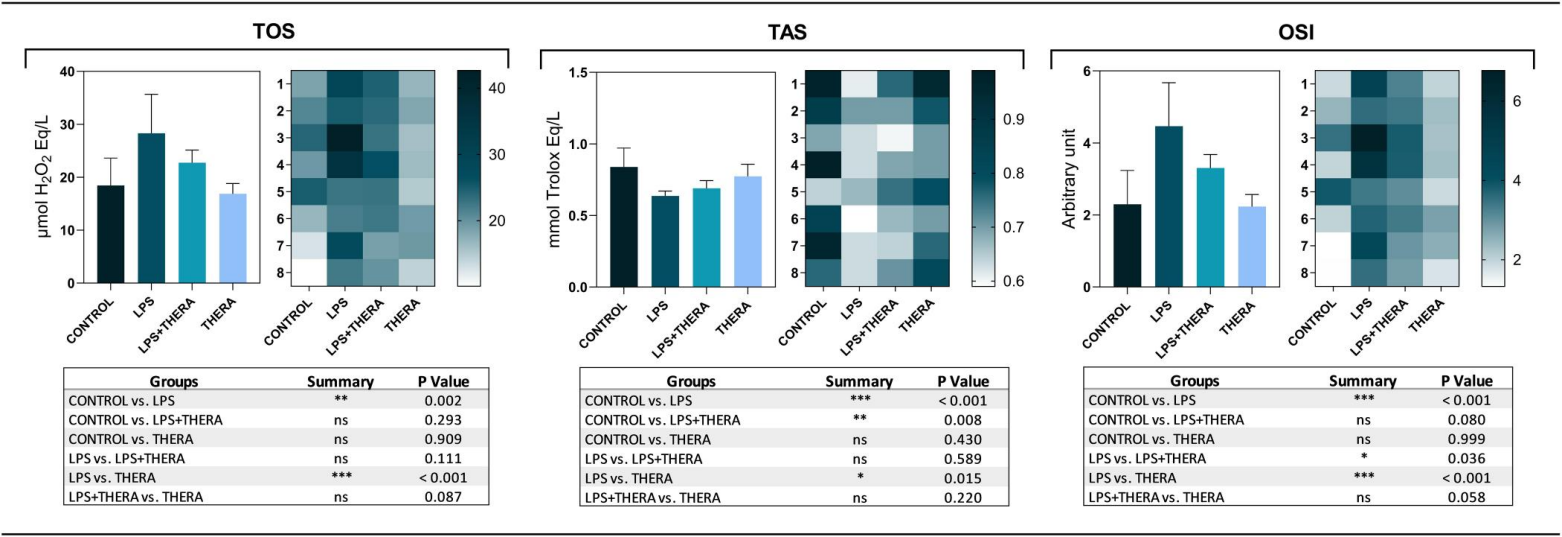
Oxidative stress parameters of heart tissues.

### 3.2 Histopathological Findings

Microscopic analysis of the aorta and heart in both the Theranekron and Control groups did not reveal any pathological alterations. The cardiomyocytes in these groups exhibited signs of elongation, branching, normal size, and well‐defined intercalated discs. Additionally, the heart cells were surrounded by a rich capillary network and delicate endomysium sheaths. In contrast, the LPS group showed changes in the heart tissue, such as endothelial sloughing in the aortas, hyperemia, hemorrhage, and infiltration of inflammatory cells. These pathological changes were ameliorated with Theranekron pretreatment (Figure 2).

**Figure 2:**
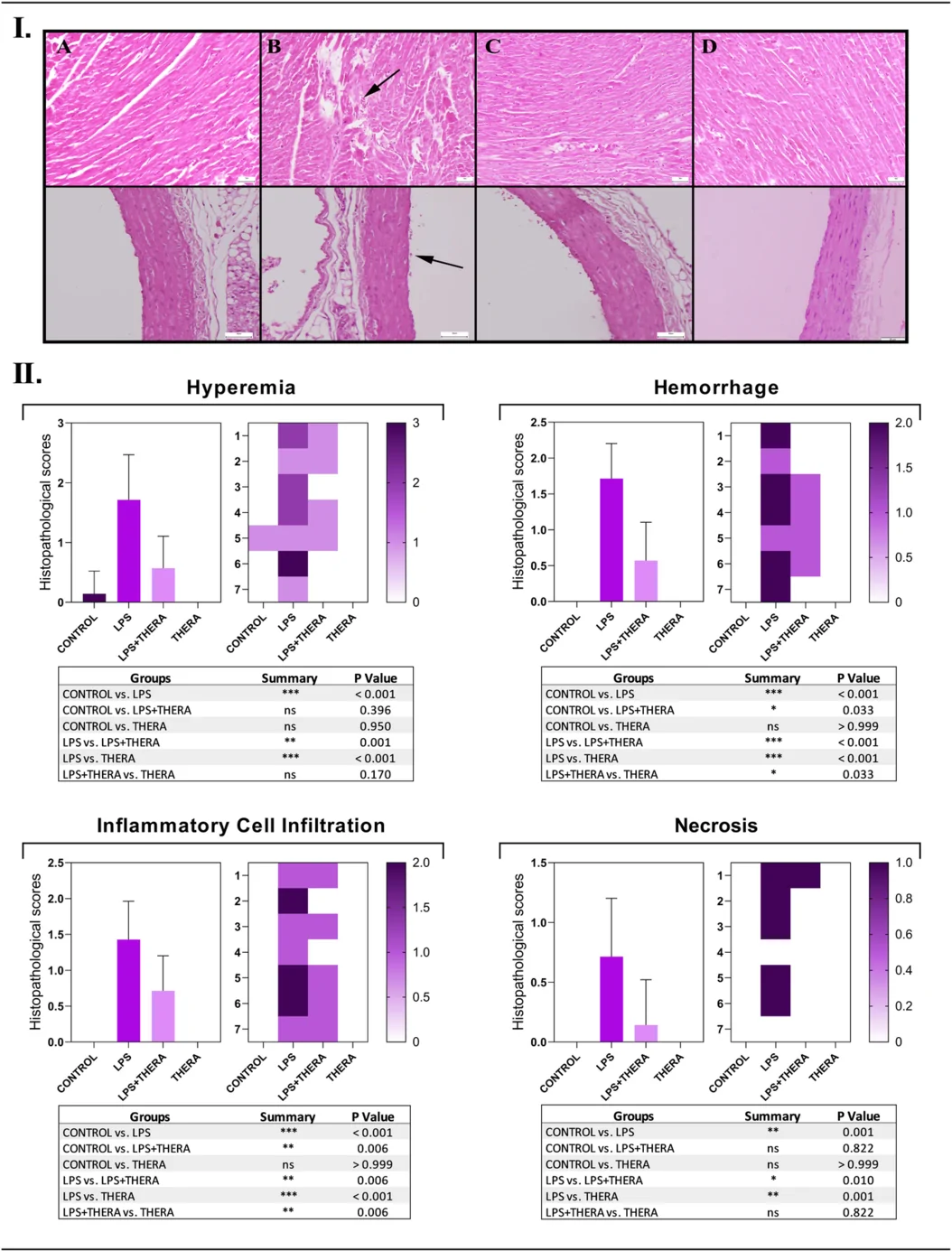
Microphotographs showing the hearts (top row) and aortas (bottom row) sections across different groups.

### 3.3 Immunohistochemical Examination

Immunohistochemical analysis showed very weak or no expression of Cas‐3, iNOS, and TNF‐α in the control group. In the LPS group, the expression of Cas‐3, iNOS, and TNF‐α increased from slight to marked levels in both the myocardium and the aortic endothelium. Pretreatment with Theranekron led to a decrease in these markers in the LPS + Theranekron group, while the Theranekron only group showed minimal or no immunoreactivity (Figures 3, 4, 5).

**Figure 3:**
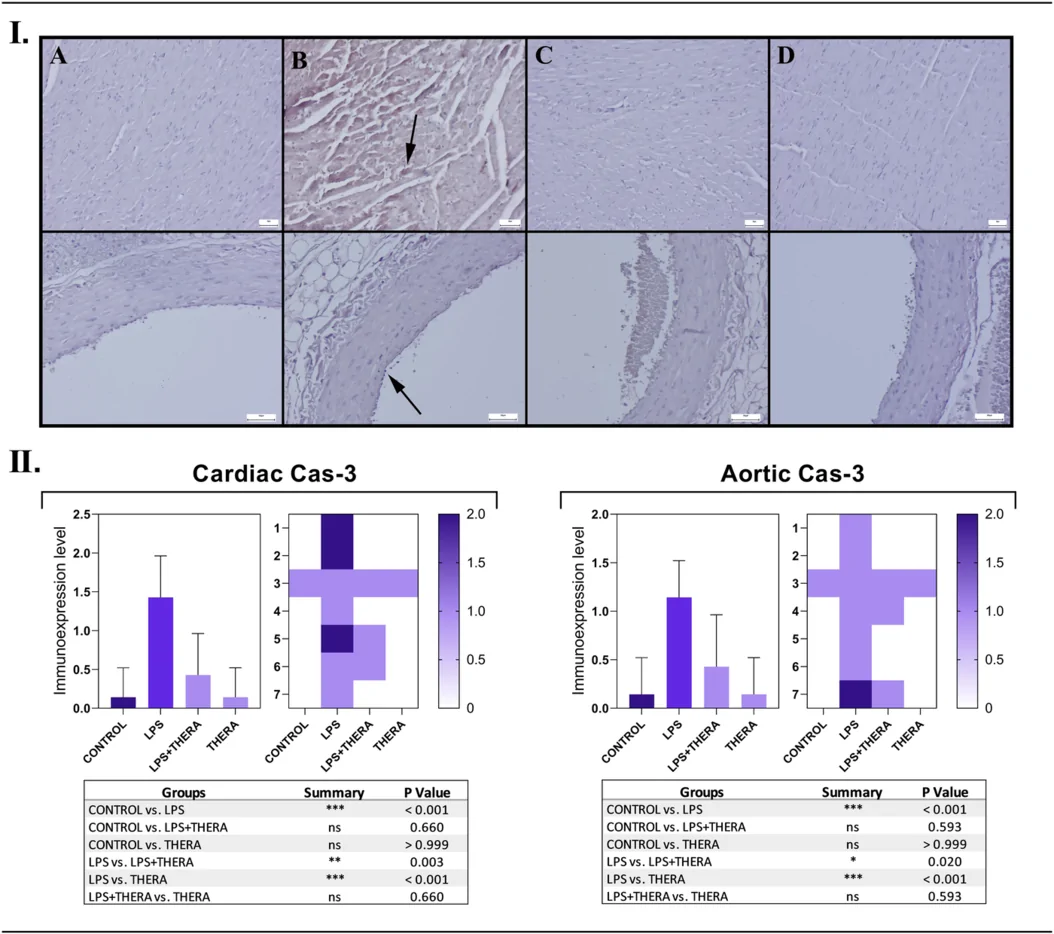
Cas‐3 IHC findings in the heart (top row) and aorta (bottom row) sections across different groups.

**Figure 4:**
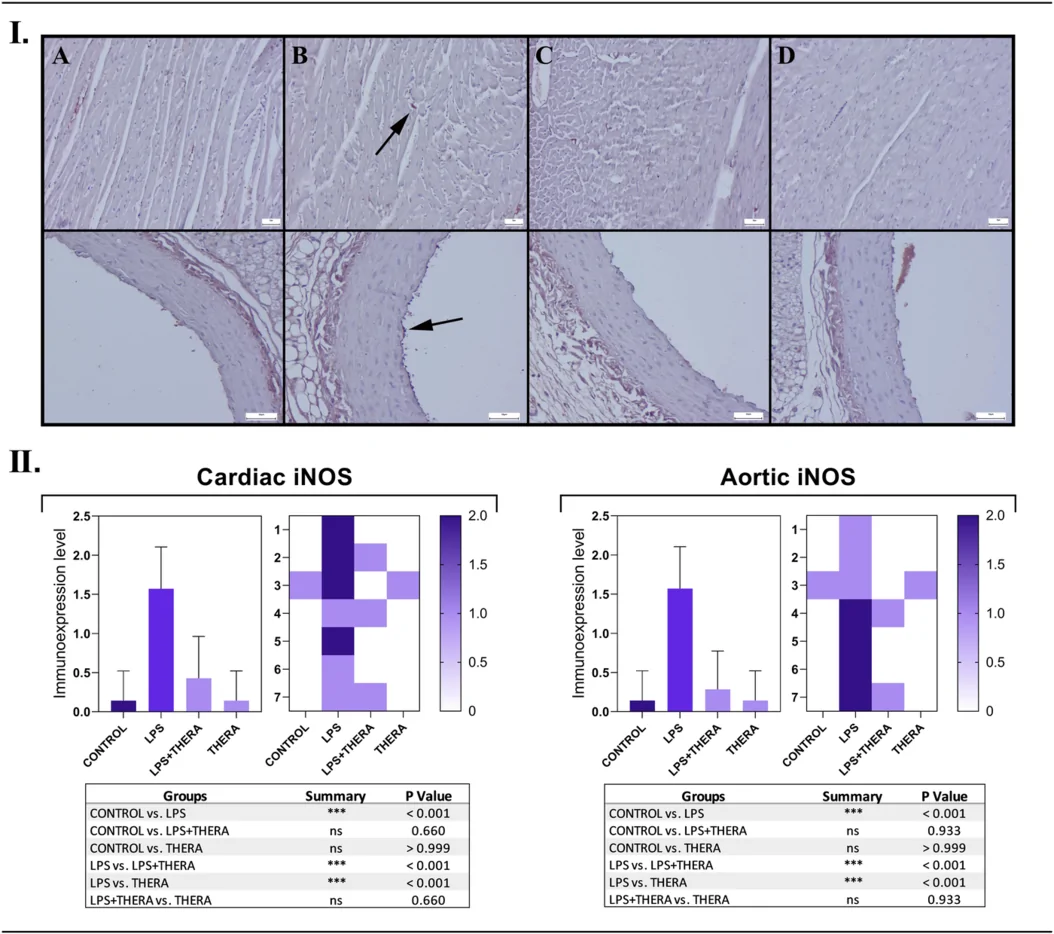
iNOS IHC findings in the heart (top row) and aorta (bottom row) sections across different groups.

**Figure 5:**
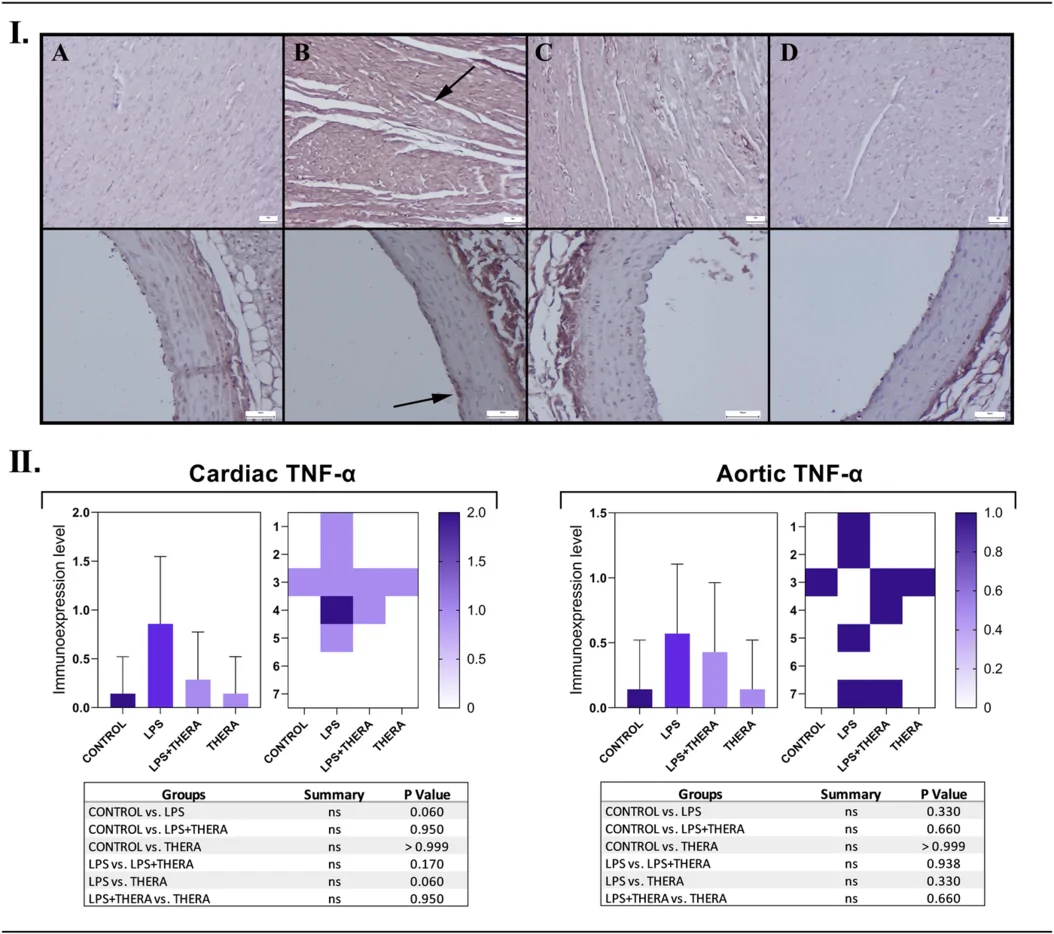
TNF‐α IHC findings in the heart (top row) and aorta (bottom row) sections across different groups.

### 3.4 Genetic Results

The expressions of IL‐6, STAT3, HIF‐1α, and VEGF, which are genes in the HIF‐1α pathway, were significantly higher in the LPS group compared to the control (*p* < 0.001 for all) and Theranekron (*p* < 0.001 for all) groups. However, the levels of all these genes were significantly lower in the LPS + Theranekron group compared to the LPS group (*p* < 0.001 for all). Additionally, STAT3 levels in the LPS + Theranekron group were significantly higher than in the Theranekron group (*p* = 0.013) (Figure 6).

**Figure 6:**
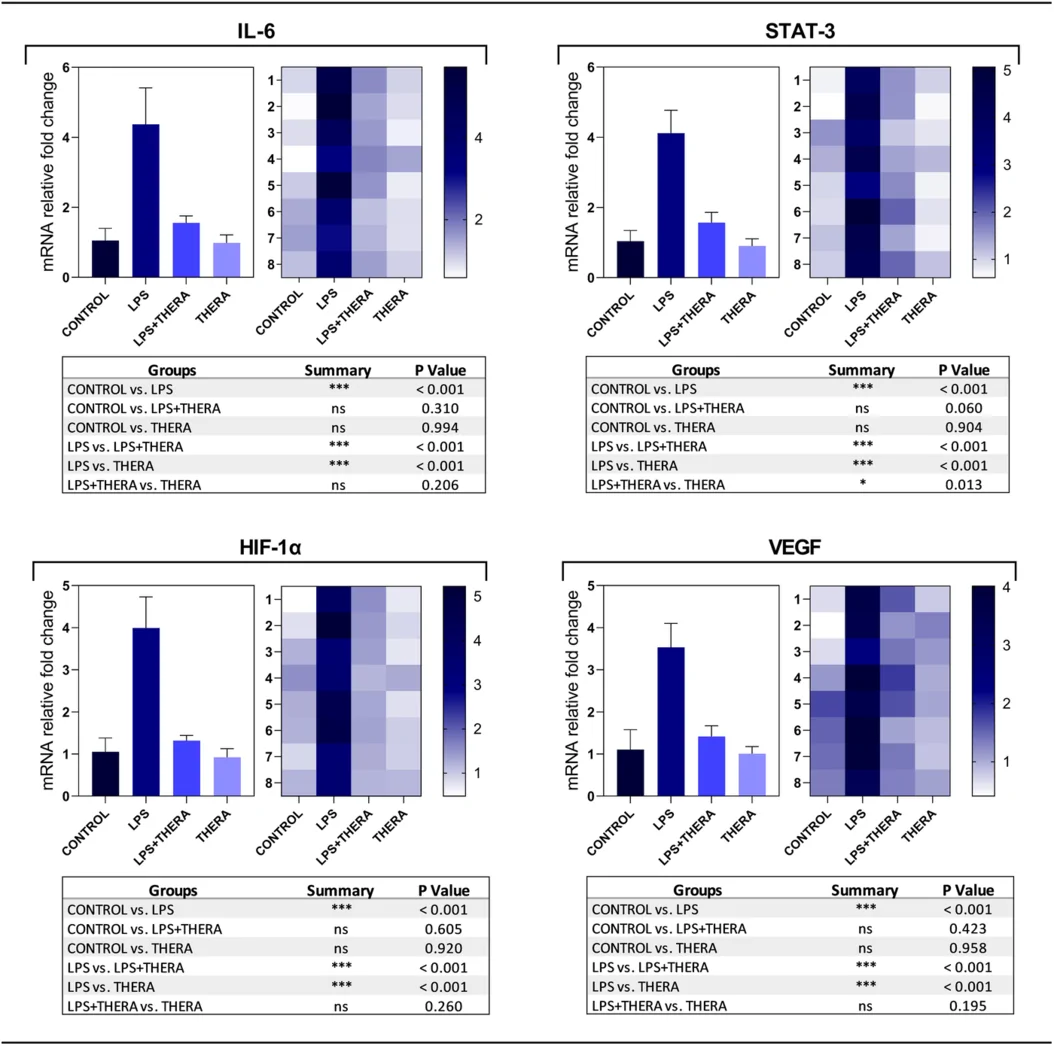
The expression levels of IL‐6, STAT3, HIF‐1α, and VEGF in cardiac tissues.

## 4 Discussion

Current studies with Theranekron, a venom derived from *T. cubensis* (family Theraphosidae), have focused on its features regarding inflammation and apoptosis. Beyond its cardiovascular effects, the clinical significance of Theranekron has been demonstrated in various noncardiovascular settings. For instance, its application in neurological injury models has shown promising outcomes in reducing oxidative stress and apoptosis, as observed in the sciatic nerve injury studies [17]. Similarly, its anti‐inflammatory properties have been effectively utilized in cystitis models, where it ameliorated TNF‐α expressions [18]. These findings highlight Theranekron's potential for broader therapeutic applications, emphasizing its versatility as a pharmacological agent.

The protection of the heart and vascular structures, which are especially affected by hypoxic conditions and all harmful pathogens in the blood due to the blood tissue it contains, is vitally important. Although many different agents have been tried in studies to protect these tissues, the lack of an ideal solution continues to result in mortality caused by cardiovascular dysfunctions. For instance, studies on sepsis patients have highlighted how systemic inflammatory responses exacerbate cardiovascular damage, as seen in the vascular endothelium and heart tissue [19]. These findings underline the need for comprehensive treatments, such as Theranekron, which has shown potential in mitigating oxidative stress and inflammation in preclinical models [13].

In LPS‐induced systemic inflammation, such damage can be observed in the vascular endothelium, which is a very naive internal structure, owing to the prooxidant and proinflammatory substances circulating in the blood as part of the systemic inflammatory response, and comparable damage can be seen diffusely in the heart tissue [19, 20]. Increased TOS and OSI values are consistent with higher levels of oxidative stress in the tissues. Oxidative stress arises from elevated oxidant molecules within the cells. The decrease observed only in the OSI values in the treatment groups may indicate a possible antioxidant‐related effect of the active substance, although this limited change might become more evident with higher or longer‐term doses. In a previous study we conducted, the positive effects of Theranekron against oxidative stress were similarly reported [13]. On the other hand, such damage mechanisms are known to trigger other processes by activating different cellular pathways [21, 22]. Theranekron pretreatment was associated with reduced OSI and lower TNF‐α immunoreactivity, which may partly reflect attenuation of inflammatory and oxidative responses in this model [13, 18]. Accordingly, single‐dose Theranekron administration partially ameliorates oxidative stress imbalance mainly by reducing the oxidant burden rather than by increasing endogenous antioxidant activity. TAS values in the Theranekron‐only group did not rise relative to the control group, supporting the view that the effect is driven by a lower oxidant load rather than enhanced antioxidant capacity.

Inflammation, one of the other main mechanisms, may occur on its own or secondary to oxidative stress, and the histopathological data, together with the iNOS and TNF‐α immunoreactivity that increased in parallel with rising TOS and OSI values, are consistent with a relationship between oxidative stress and inflammation. Hyperemia, hemorrhage, inflammatory cell infiltration, and endothelial sloughing in both cardiovascular tissues suggest that prooxidant and proinflammatory substances trigger a pronounced inflammatory response. The reduction of TNF‐α immunoreactivity, which is known to increase in acute inflammatory conditions, and in particular of neutrophilic infiltration associated with Theranekron pretreatment suggests an acute anti‐inflammatory association [23]. Akdağ and colleagues reported in their cystitis model that increased TNF‐α expression was ameliorated with Theranekron pretreatment [18]. It has been reported that not only toxic substances but also many natural bioactive compounds reduce inflammatory cell infiltration and inflammatory damage in various physiological systems [24]. In addition, the reduction in OSI observed with Theranekron pretreatment may be associated with the attenuation of inflammatory responses, suggesting a possible interaction between inflammation and oxidative stress in this model.

The impairment of nitric oxide (NO) synthesis—produced by the vascular endothelium and known to play a critical role in vascular regulation—due to endothelial damage may lead to reduced vascularization of distal tissues, ultimately resulting in tissue hypoxia [25]. From this point of view, cardiac damage, which is secondary to endothelial damage in cardiac tissue or increases with the direct effect of harmful substances circulating in the blood, may gradually increase [26].

Proinflammatory cytokines and reactive oxygen species can stabilize HIF‐1α under normoxia, and the HIF‐1α axis is increasingly regarded as a node that couples inflammatory and hypoxic signaling [27, 28]. This coupling is well characterized upstream: in immune cells, lipopolysaccharide raises HIF‐1α through TLR4/NF‐κB–dependent normoxic stabilization rather than through hypoxia alone [29]. HIF‐1α, in turn, participates in a feed‐forward relationship with IL‐6/STAT3 signaling and promotes VEGF expression [5, 6, 7, 12], and interrupting upstream STAT3 signaling has been shown to lower both HIF‐1 and VEGF in other, cancer‐cell systems [30]. Consistent with a shared inflammatory–hypoxic program, HIF‐1α is inflammation‐induced and interfaces with NF‐κB, JAK2/STAT3, and VEGF signaling in sepsis [31], whereas reactive oxygen species act as proximal stabilizers of HIF‐1α in myocardial ischemia–reperfusion [32]. Against this background, the concordant increase in IL‐6, STAT3, HIF‐1α, and VEGF mRNA in the LPS group, together with their parallel reduction after Theranekron pretreatment, is consistent with attenuation of this inflammatory–hypoxic transcriptional program. This effect may be attributable either to a direct effect of Theranekron on HIF‐1α stabilization or transcriptional activity, or to its ability to reduce OSI and inflammatory immunoreactivity. However, our current data are more consistent with indirect downregulation of HIF‐1α‐related transcription rather than direct pathway inhibition. Further pathway‐specific studies are needed to clarify this mechanism.

In the event of apoptosis or necrosis, which is the ultimate condition created by these mechanisms at the cellular level in case of damage, the death of the cell occurs and is irreversible [33]. It is very important for survival to prevent these consequences with the intervention of drugs before the development of damage. When Cas‐3 activity, one of the immunoexpressions of the study, is examined, it can be said that apoptosis is activated in the damaged heart and aorta tissues. Considering the effects of Theranekron, which shows apoptosis‐inhibitory behavior, it can be said that it does this by regressing inflammation. Similar to these results, Theranekron has also been shown to reduce Cas‐3 levels in the sciatic nerve injury model conducted by Sencar et al. [17].

The present study should be interpreted within the context of an acute pretreatment model. Accordingly, the findings suggest that Theranekron pretreatment may be associated with attenuation of early cardiovascular injury markers, but they do not demonstrate therapeutic efficacy when administered after injury onset. This distinction is important because prophylactic protection in an acute endotoxemia model cannot be directly extrapolated to the clinical treatment of established sepsis‐related cardiovascular dysfunction. Future studies using post‐treatment protocols and functional cardiovascular assessments are needed to determine whether Theranekron has therapeutic relevance beyond early experimental protection.

Several limitations should be considered when interpreting the mechanistic findings of this study. First, the HIF‐1α axis was assessed at the mRNA level only, without protein‐level validation, nuclear localization analysis, or pathway‐specific functional interventions. Second, because the in vivo phase had been completed under the approved protocol, a separate ethanol‐equivalent solvent‐control group was not included, and the contribution of the excipient cannot therefore be fully excluded. Third, the study used a single dose and a single time point, without dose–response or multi‐time‐point evaluation. Fourth, cell‐specific assays distinguishing cardiomyocyte from vascular endothelial effects were not performed, so the cellular target of the observed effects remains undefined. Finally, Theranekron is a commercial veterinary preparation containing a complex *T. cubensis* alcoholic extract rather than a purified active compound; the findings should therefore not be interpreted as evidence of a specific molecular target, and future studies using chemically characterized fractions or purified components are required to clarify the pharmacological basis of its effects.

## 5 Conclusions

In conclusion, Theranekron pretreatment was associated with partial attenuation of early LPS‐induced cardiovascular injury. These effects were accompanied by reductions in oxidative stress imbalance, inflammatory and apoptotic immunoreactivity, and in LPS‐induced IL‐6, STAT3, HIF‐1α, and VEGF mRNA upregulation. However, the lack of solvent‐control, protein‐level validation, dose‐response, multi‐time‐point, and cell‐specific experiments limits causal mechanistic interpretation, and further studies incorporating protein expression analyses, localization assays, and targeted pathway modulation are required.

## Author Contributions

**Mehtap Savran:** conceptualization, methodology, data curation, validation, writing – original draft, investigation, project administration. **Halil Asci:** conceptualization, methodology, software, validation, formal analysis, supervision, funding acquisition, writing – review and editing, writing – original draft. **Orhan Imeci:** conceptualization, methodology, software, data curation, investigation, formal analysis, writing – original draft, resources. **Furkan Cagri Oguzlar:** methodology, software, formal analysis, visualization, writing – review and editing. **Ozlem Ozmen:** methodology, data curation, formal analysis, validation, visualization, supervision. All authors commented on previous versions of the manuscript. All authors read and approved the final manuscript.

## Ethics Statement

The methodology conducted on animals was reviewed and approved by the Experimental Animals Local Ethics Board of Suleyman Demirel University (Approval No: 11.05.2023‐168/05) as it adhered to the ARRIVE 2.0 guidelines.

## Conflicts of Interest

The authors declare no conflicts of interest.

## Data Availability

The data that support the findings of this study are available from the corresponding author upon reasonable request.
